# Relationship Between COVID-19 Related Knowledge and Anxiety Among University Students: Exploring the Moderating Roles of School Climate and Coping Strategies

**DOI:** 10.3389/fpsyg.2022.820288

**Published:** 2022-03-30

**Authors:** Frank Quansah, John E. Hagan, Francis Ankomah, Medina Srem-Sai, James B. Frimpong, Francis Sambah, Thomas Schack

**Affiliations:** ^1^Department of Educational Foundations, University of Education, Winneba, Ghana; ^2^Department of Health, Physical Education and Recreation, University of Cape Coast, Cape Coast, Ghana; ^3^Neurocognition and Action-Biomechanics-Research Group, Faculty of Psychology and Sports Science, Bielefeld University, Bielefeld, Germany; ^4^Department of Education and Psychology, University of Cape Coast, Cape Coast, Ghana; ^5^Department of Education, SDA College of Education, Asokore-Koforidua, Ghana; ^6^Department of Health, Physical Education, Recreation and Sports, University of Education, Winneba, Ghana

**Keywords:** anxiety, coping strategies, COVID-19 pandemic, Ghana, knowledge, school climate

## Abstract

The emergence of the COVID-19 pandemic resulted in abrupt disruptions in teaching and learning activities in higher education, with students from diverse programs suffering varying levels of anxieties. The physical education field happens to be one of the most affected academic areas due to its experiential content as a medium of instruction. In this study, we investigated the roles of school climate and coping strategies in the relationship between COVID-19 related knowledge and anxiety. Through the census approach, a cross-sectional sample of 760 students was administered a questionnaire in two universities offering Physical Education in Ghana: the University of Education, Winneba, and University of Cape Coast. The outcome of the study found a positive and significant link between COVID-19 knowledge and anxiety. Further, school climate and coping strategies significantly moderated the relationship between students’ COVID-19 knowledge and associated anxiety. The findings have implications for creating a conducive school environment that reduces the risk of COVID-19 infection and through students’ adoption of active coping strategies in an attempt to reduce psychological distress associated with COVID-19 anxiety.

## Introduction

The coronavirus (COVID-19) pandemic has caused significant public health concerns and altered peoples’ behaviors. The pandemic impacted variedly on human behavior, emotions, and cognition, eliciting diverse behavioral and psychological responses linked to the disease that have caused mental health-related issues across many societies (Alsukah et al., 2020; Clay and Parker, 2020; Lee and You, 2020; Lei et al., 2020). These manifestations of the pandemic have caused feelings of anxiety in many spheres of daily life, including health, work, school, and relationships (Germani et al., 2020a,b; Gong et al., 2020). Previous research has shown the negative effect of anxiety on mental health and psychological well-being (Huang and Zhao, 2020; Ornell et al., 2020; Torales et al., 2020). The low predictability of COVID-19 consequences may affect people’s mental health, due to their inability to adequately handle their current situation, especially on matters related to anxiety management (Jeong et al., 2016; Cao et al., 2020; Li et al., 2020a,b,c; Wang et al., 2020). According to Shigemura et al. (2020), fear and insecurity increase anxiety levels in healthy individuals and build up related pre-existing symptoms.

Given the devastating COVID-19 effects worldwide, having knowledge and complying with the virus’ preventive and management protocols might be key toward lessening the psychological burden associated with the pandemic (Wang et al., 2021). Empirical evidence has already shown that during public health crises, adequate knowledge during an epidemic or disease-related literacy of people in any given population plays a central role in the adoption of preventive behaviors among the general population and eventually alleviate mental health challenges (Brug et al., 2004; Sadique et al., 2007; Yıldırım et al., 2020). For example, knowledge has been shown to be associated with actual knowledge (Flynn and Goldsmith, 1999; Krawczyk et al., 2013), psychological distress (Wang et al., 2020; Wolf et al., 2020), boost confidence in a person’s knowledge, which in turn, triggers more information search, adoption of preventative behaviors, and promotes decision-making process during disease outbreak (Park et al., 1988; Krawczyk et al., 2013). Other studies have also shown that knowledge can help change unfavorable attitudes and behaviors, thus, may successfully curb infectious diseases and epidemics (Verelst et al., 2016). Recent research has implied that a lack of knowledge about the COVID-19 pandemic and insufficient preventive behaviors, impacted the general mental health (Yildirim and Güler, 2020), and heightened anxiety experiences among several individuals (Du et al., 2020; Wang et al., 2020; Zhai and Du, 2020).

The school climate is one environment that has significantly been wrecked by the nature of the pandemic globally through interruptions in academic work by schools’ closure and/cancelation of academic-related activities due to stringent precautionary restrictions (e.g., physical or social distancing measures) (Mandapat and Farin, 2021). School climate, in the context of this research, reflects the extent to which students are confident and perceive the teaching and learning environment to be safe from COVID-19 infection. Research works have established the link between school climate and psychological variables such as anxiety and stress (Germani et al., 2020b; Lin et al., 2020; Li and Lyu, 2021; Šrol et al., 2021; Quansah et al., 2022). Šrol et al. (2021), for example, found in their study that students experienced heightened levels of anxiety when they do not trust that their institution can provide a safe learning environment for them during the COVID-19 pandemic. Lin et al. (2020) also found that persons who perceived their environment as safe and less susceptible to contracting COVID-19, experienced low levels of anxiety. This situation creates an enormous burden on parents, students, educators, educational institutions, and governments to safeguard the continuance of learning and at the same time protect them from contracting the virus (UNESCO, 2020; Laar et al., 2021).

While facing these unprecedented emergencies and institutions striving to enhance the safety of the school environment (i.e., positive school climate), students could rely on their personal resources to cope with events they may perceive as challenging or threatening (Peters and McEwen, 2015; Peters et al., 2017; Alsukah et al., 2020). Prior research has shown that coping strategies of persons are related to their level of anxiety and stress (see Main et al., 2011; Lyons et al., 2016; Iddi et al., 2021; Li et al., 2021). Li et al. (2021) discovered that students who adopted coping strategies which focused their attention on solving the problem experienced lower levels of psychological distress. Iddi et al. (2021) also revealed that individuals who were concerned about their safety amidst COVID-19 coped very well. Other studies have also shown that religious coping was associated with COVID-19 related anxiety (Chow et al., 2021; Yıldırım et al., 2021; Zarrouq et al., 2021). From the psychological resilience theory perspective, individuals can successfully cope with events perceived as stressful and preserve their psychological function even in adversity, because internal and external protective strategies can help ease the negative impact of these challenging events (Main et al., 2011; Luthar et al., 2015; Lyons et al., 2016).

In the context of higher education, several universities across the world postponed or canceled campus-related activities (e.g., suspension of onsite teaching, cancelation of examinations). Consequently, teaching and learning processes had to adapt to the new reality and still offer services that promote students’ educational aspirations, academic engagement and achievement, while minimizing dropouts (Capone et al., 2020). This unanticipated emergency and the unparalleled severe precautionary measures remarkably placed everyone involved in the educational system at risk in terms of mental health and psychosocial functioning (Owen et al., 2018; Capone et al., 2020). For example, a university’s organizational structure related to intermittent changes in examination schedules, due dates of specific tasks, and projects as well as cancelation of lessons have been found to elicit anxiety-related challenges among students (Mancini et al., 1983; Pfeiffer, 2001). Hence, it is reasonable to anticipate that COVID-19 related emergencies and associated mitigation strategies may generate anxiety and other mental health challenges on students’ overall well-being (Alsukah et al., 2020; Capone et al., 2020; UNESCO, 2020; Laar et al., 2021; Wang et al., 2021). Besides, investigating university students in the Physical Education field whose academic program has been one of the most affected subjects during the pandemic is necessary because of its experiential content as a medium of instruction has curriculum implications (Lander et al., 2017). Students in this program are required to engage in practical lessons which becomes a challenge, and thus, the need for institutions to develop new strategies that allow the practical sessions to hold but in a safe and protected manner (Chaliès et al., 2008; Nash, 2010; Hume and Berry, 2013; Chen et al., 2020; Isidori, 2020; Medina and Bohórquez, 2020). But this instructional delivery mode raises several questions regarding the safety of the students from contracting the virus and their anxiety responses to such situations.

In contemporary times, research connecting students’ COVID-19 related knowledge with anxiety-related experiences using school climate and coping strategies as proxies toward active adaptation to maintain optimal psychological health in times of emergency is of great relevance. Although several studies have established the link between COVID-19 related knowledge and associated anxiety response, the findings are inconclusive. Whereas some studies have revealed negative relationship (see Du et al., 2020; Wang et al., 2020; Zhai and Du, 2020), others have found a positive relationship (see Wolf et al., 2020) as well as no relationship (see Lin et al., 2020). This inconsistency is primarily so because other variables such as school climate and coping strategies have the potency to distort the relationship (see Germani et al., 2020b; Lin et al., 2020; Iddi et al., 2021; Li and Lyu, 2021; Li et al., 2021; Šrol et al., 2021). Besides, no study till date has explicitly tested these moderation effects and concurrently measured these research variables in one research design. Up until now, research linking students’ COVID-19 knowledge with anxiety-related experiences while moderating the school climate and coping strategies in Ghana is uncharted. The assumption is that school climate and coping strategies could be significant moderators of the relationship between COVID-19 knowledge and anxiety. Conducting this investigation is significant because it would provide a better insight to address students’ knowledge gaps relating to the disease, thus helping in the control of the ongoing pandemic. Findings would also help guide interventions aimed at protecting students’ mental health and overall well-being during this unprecedented era. The rationale of this study was to investigate Physical Education students within the context of the COVID-19 pandemic, with the following specific objectives:

1. Examine the influence of COVID-19 related knowledge on anxiety response.
2. Moderate school climate in the link between COVID-19 knowledge and related anxiety.
3. Moderate coping strategies in the link between COVID-19 knowledge and related anxiety.

## Materials and Methods

### Participants’ Selection

The target population was 1,003 Physical Education students; 906 from the University of Education, Winneba (UEW) (90.3%) and 97 from the University of Cape Coast (UCC) (9.7%) in Ghana. These two public universities were used because they are the only universities offering the conventional Physical Education program in Ghana spanning over three decades. The census approach was used to target all the respondents (i.e., students) in the population. Employing the descriptive cross-sectional survey design, a total of 760 Physical Education students participated in the present study from the two selected public Universities from the Department of Health, Physical Education, Recreation and Sports (HPERS) of UEW and the Department of Health, Physical Education and Recreation (HPER), UCC. Out of the total sample, 86.1% (*n* = 668) of the participants were recruited from UEW, whilst 12.1% (*n* = 92) were recruited from UCC. These students were all regular Physical Education students without any specialization in the above-mentioned universities.

The sample size comprised 67.1% Christians (*n* = 510), 26.6% Muslims (*n* = 202), 5.5% traditionalists (*n* = 42) and 6 atheists (0.80%). The males constituted about 74.5% (*n* = 566) of the sample and 26.5% represented females (*n* = 194). About 35.3% (*n* = 268) were within the age ranges between 20 and 24 years, 16.3% (*n* = 124) fell within the age category of 25 and 29 years. Two hundred and forty-six participants representing 32.4% were between 30 and 34 years whilst 3.4% (*n* = 26) and 12.6% (*n* = 96) were in the age categories of 35–39, and 40 years and above respectively. The original number of participants expected was 1,003; however, 106 of the responses were not valid (non-response for over 70% of the items) whereas 137 of the participants either dropped out or opted not to be part of the study. The overall response rate was 75.8%.

### Study Variables

#### Outcome Variable

COVID-19 related anxiety was the outcome variable for this study. The variable was conceptualized as the extent to which students have been bothered with the issue of COVID-19 during the past month, including the day of the data collection exercise. A scale point ranging from 0 to 3 was adopted as a response option, with 0 being “*not at all*,” 1 representing “*somewhat*,” 2 being “*moderate*,” and 3 representing “*very much so.*” The scale was a six-item unidimensional instrument. The items include: “*I feel unsteady*,” “*I feel nervous*,” and “*I fear the worst happening.*” The reliability of the scale was tested using Omega ω, yielding a coefficient of 0.713 and factor loadings ranging from 0.621 to 0.812. For interpretation purposes, a mean score greater than 1.5 showed a moderate to a high level of COVID-19 related anxiety whereas a mean score less than 1.5 depicted a low to no existence of COVID-19 related anxiety. The items measuring anxiety were adapted from the non-clinical symptoms aspect of the anxiety scale by Beck et al. (1988). The fit indices as shown by GFI = 0.92, CFI = 0.90; RMSEA = 0.06; and CMIN = 3.1 indicated a good fit. The factors loadings were also above 0.72. The survey was administered in the English language.

Omega reliability instead of Cronbach alpha was used to estimate the internal consistency of the anxiety scale. This choice was because the Omega reliability estimation procedure overcomes the numerous limitations of Cronbach alpha. Cronbach alpha is based on the tau-equivalence assumption which is difficult or never met. Omega, however, rely on congeneric assumption. Also, alpha provides a lower bound to the reliability which can be misleading. While alpha estimates are based on variance estimates of items, omega uses item loadings (Sijtsma, 2009; Dunn et al., 2014; DeVellis, 2017; Hayes and Coutts, 2020). Based on these limitations, among others, omega reliability is recommended. It is instructive to indicate that Omega was also used for other measures (such as the coping inventory) used in this study which were Likert scale in nature.

#### Predictor Variable

The predictor variable for this research was knowledge about COVID-19. Six questions were posed to the participants regarding the symptoms, transmission of the disease, treatment, and management of COVID-19. Examples of items are: “*What are the main ways in which people are currently getting infected with the new coronavirus?*,” and “*Do health measures such as early case detection and isolation, contact tracing and isolation, and social distancing help reduce the spread of COVID-19?*” For all the items, options were provided for responses to be selected by the participants and the total score was interpreted out of a score of 6. Kuder–Richardson 21 estimation procedure was used to estimate the reliability of the item responses. A reliability estimate of 0.73 was obtained. Kuder–Richardson 21 was used because the items on COVID-19 knowledge were scored dichotomously as ‘1’ or ‘0’ for right or wrong, respectively (Nitko, 2001; Allen and Yen, 2002; Crocker and Algina, 2008).

#### Moderating Variables

The study used two moderating variables: coping strategies and school climate. The coping strategy in this study is conceptualized as the reactions of participants when they found themselves in a stressful situation induced by the COVID-19 pandemic. The coping strategy instrument was a 16-item multidimensional scale with four sub-scales: Active coping, Religious coping, Behavioral disengagement, and Emotional support. The sub-dimensions had four items each and the responses were measured using a scale of 1 to 4, with 1 being “*not adopted*,” 2 being “*somewhat or moderately adopted*,” 3 representing “*much adopted*” and 4 being “*very much adopted.*” The Omega ω reliability estimate yielded the following coefficients: active coping-0.705 (loadings ranging from 0.546 to 0.748); religious coping-0.778 (loadings from 0.661 to 0.841); behavioral disengagement-0.703 (loadings from 0.561 to 0.776); and emotional support-0.701 (loadings from 0.50 to 0.714). The coping scale was adopted from Quansah et al. (in press). The fit indices and factor loadings of the coping scale as validated in this study are acceptable.

School climate was the second moderating variable for the study. School climate was conceptualized as the extent to which students are safe from contracting the COVID-19 virus. Five items were posed to the participants which include: “*Do you feel confident participating in practical lessons during the COVID-19 outbreak?*,” “*Are the necessary protective equipment available for practical lessons amidst the outbreak of COVID-19?*,” and “*Are you comfortable participating in teaching and learning activities amidst this COVID-19 outbreak?*” All the items were measured using dichotomous response, “Yes or No.” All “yes” responses were given a numerical weight of 1 and all the “no” responses were assigned a value of 0. The total score is summed out of 5. At least a score of 4 is interpreted as a positive school climate (i.e., a score of 4–5); whereas, the school climate is considered as negative with scores ranging from 0 to 3. Therefore, a positive school climate is highly safe in terms of contracting COVID-19. The Kuder–Richardson 21 reliability estimate yielded a coefficient of 0.718. The scale for school climate was scored dichotomously, therefore, Kuder–Richardson 21 was used (Nitko, 2001; Allen and Yen, 2002; Crocker and Algina, 2008; Quansah, 2017). The fit indices are shown by GFI = 0.94, CFI = 0.91; RMSEA = 0.08; and CMIN = 2.5. In addition, all the factor loadings were above 0.70.

### Data Collection Procedure

The study obtained ethical clearance from the Institutional Review Board (IRB) of the University of Cape Coast, Ghana with reference number: UCCIRB/EXT/2020/25. After the IRB approval, permission was sought from the Head of Departments, HPERS and HPER. The data were gathered from the lecture halls of the university after making arrangements with the lecturers. The data administration was done by the researchers. Prior to the administration, the researchers established a good rapport with the participants during which the participants were given a detailed briefing regarding the purpose of the work and the need to participate in this research. Participants were informed that only the researchers would have access to their responses. Further, participants were assured that their responses would be kept confidential and that to ensure anonymity, their names would not be needed on the survey instrument. Participants were also informed that their participation was strictly voluntary and that at any point in time, they could decide to either withdraw or discontinue with the survey without any consequences. Written consent was obtained from each participant. Responding to the instrument took about 20 minutes. The entire data collection lasted for 1 month. Throughout the data collection period, both the researchers and participants were protected, as all the COVID-19 safety protocols were adhered to. Both researchers and participants wore nose/face masks, frequent hand washing, and sanitizing were done, including the observation of social distancing.

### Data Analysis Strategy

The data were screened for outliers and missing cases with none found using SPSS software version 25. Descriptive analysis was conducted to understand the nature of variables of the study and inspect the relationship existing between the variables. Hence, correlation analysis, means and standard deviations of the variables were computed. Simple linear regression, with 5,000 bootstrap samples, was also performed to investigate the relationship between COVID-19 knowledge and anxiety experienced. A simple moderation analysis was conducted to examine the moderating effect of school climate in the relationship between COVID-19 knowledge and anxiety experienced. Model 1 of PROCESS macro for SPSS by Hayes was used for the simple moderation analysis. A four-way moderated moderation analysis, with 10,000 bootstrap samples, was performed to investigate the moderating role of coping strategies in the relationship between COVID-19 knowledge and anxiety. Apart from the 4 parallel moderators, 10 other interaction moderators were identified by default and their effects were tested. The 10 other interaction moderators have to do with the pairwise, three-way, and/or four-way interactions between COVID-19 knowledge and/or between the coping strategies. Regarding the interpretation of the moderating effect, the bootstrap confidence intervals were used. Model 3 of Hayes PROCESS macro was used for the moderated moderation analysis (Hayes, 2018).

## Results

### Descriptive Statistics

The descriptive statistics of the variables are presented in Table 1, including the correlations between the variables, their means and standard deviations.

**TABLE 1:** Correlations among variables, means, and standard deviations.

Variables	AXT	KNG	SHC	ACP	RCP	BDC	ESS
Anxiety (AXT)	1						
Knowledge (KNG)	0.110**	1					
School climate (SHC)	−0.139**	0.043	1				
Active coping (ACP)	0.168**	0.317**	–0.047	1			
Religious coping (RCP)	0.018	0.243**	−0.075*	0.386**	1		
Behavior disengagement (BDC)	0.137**	–0.049	0.089*	0.147**	0.198**	1	
Emotional support (ESS)	0.201**	0.223**	–0.009	0.409**	0.330**	0.309**	1
Mean	1.576	4.345	2.421	2.650	2.789	1.828	2.50
SD	0.623	1.219	1.495	0.746	0.852	0.731	0.697

*Correlation is significant at the 0.05 level (2-tailed).

**Correlation is significant at the 0.01 level (2-tailed).

As presented in Table 1, anxiety was found to be positively related to knowledge on COVID-19 (*r* = 0.110) and three dimensions of coping mechanism (i.e., active coping-0.168; behavioral disengagement-0.137; and emotional support-0.201). Religious coping was not related to COVID-19 related anxiety, however school climate had a negative relationship with COVID-19 related anxiety (*r* = −0.139). The four dimensions of coping strategies were significantly related to one another, with correlation coefficients ranging between 0.147 and 0.409. Except for school climate and behavioral disengagement, knowledge on COVID-19 was positively related to active coping (*r* = 0.317), religious coping (*r* = 0.243), and emotional support (*r* = 0.223).

Further, COVID-19 related anxiety was moderate among the students (*M* = 1.576, *SD* = 0.623). Knowledge of COVID-19 was also found to be quite high with a mean of 4.345 (out of six items) and a standard deviation of 1.219. School climate during COVID-19 was somewhat not positive (*M* = 2.421, *SD* = 1.495). Active coping (*M* = 2.650, *SD* = 0.746), religious coping (*M* = 2.789, *SD* = 0.852), and emotional support (*M* = 2.50, *SD* = 0.697) strategies were moderately adopted by the students during the COVID-19 pandemic.

### The Influence of COVID-19 Related Knowledge on Anxiety

The details of the results are shown in Table 2.

**TABLE 2:** Model summary and regression coefficients.

Model		Sum of squares	*df*	Mean square	*F*	Sig.	*R* ^2^	*SE*
1	Regression	3.558	1	3.558	9.266	0.002	0.012	0.620
	Residual	291.016	758	0.384				
	Total	294.574	759					

		***B***	***SE***	**Beta**	***t***	**Sig.**	**LLCI**	**ULCI**

1	(Constant)	1.332	0.083		16.002	0.000	1.169	1.496
	Knowledge	0.056	0.018	0.110	3.044	0.002	0.020	0.092

*Outcome variable: COVID-19 related anxiety.*

*Predictors: (constant), knowledge on COVID-19.*

The overall model, with COVID-19 knowledge as a predictor and COVID-19 related anxiety as a criterion, is significant, *F*(1,758) = 9.266, *p* = 0.002. It was found that about 1.2% of the variations in COVID-19 related anxiety is explained by knowledge of COVID-19. The results further indicate that knowledge significantly and positively predicted COVID-19 related anxiety, *B* = 0.056, *t* = 3.044, *CI* (0.020, 0.092).

### Moderating Role of School Climate in the Link Between COVID-19 Related Knowledge and Anxiety

The study also had the objective of establishing the role of COVID-19 knowledge and COVID-19 related anxiety response. The details of the results are presented in Tables 3, 4.

**TABLE 3:** Interaction of school climate between COVID-19 knowledge and anxiety response.

Model	*B*	*SE*	*t*	LLCI	ULCI
Constant	–0.021	0.318	–0.065	–0.645	0.604
Knowledge (KNG)	0.292	0.070	4.161	0.155	0.430
School climate (SHC)	0.792	0.180	4.397	0.438	1.145
KNG*SHC (moderator)	–0.138	0.040	–3.470	–0.216	–0.060

Model summary: R^2^ = 0.047, F(3,756) = 12.341, p < 0.001; R^2^ change [KNG*SHC]: R^2^ = 0.0152, F(1,756) = 12.039, p = 0.001; outcome variable: anxiety.

**TABLE 4:** Probing the moderating effect of school climate.

Conditional effect							Data list sample		
SHC	Effect	*SE*	*t*	*p*	LLCI	ULCI	SHC	KNG	Anxiety
Positive	−0.154	0.034	−4.614	0.000	−0.229	−0.015	Positive	5	1.23
Negative	0.016	0.022	0.747	0.455	−0.026	0.059	Negative	5	1.64

The results found that knowledge, school climate, and the interaction of knowledge and school climate explain about 4.7% variability in COVID-19 related anxiety. For the moderation analysis, school climate was categorized as positive and negative, where negative school climate was used as the reference group. The moderator school climate during COVID-19 significantly contributed 1.52% variations to COVID-19 anxiety, *F*(1,756) = 12.039, *p* = 0.001. Overall, school climate significantly and negatively moderated the relationship between COVID-19 knowledge and anxiety, *B* = −0.138, *SE* = 0.040, *Boot CI* (−0.216, −0.060).

The significant moderating effect was probed to understand the nature of the moderation (see Table 4).

The probing results, as shown in Table 4, indicated that the presence of positive school climate distorts and significantly changes the direction of the relationship between students’ COVID-19 knowledge and their anxiety responses, *B* = −0.154, *SE* = 0.034, *BootCI* (−0.229, −0.015). Negative school climate, however, did not show any evidence of change in the relationship between students’ COVID-19 knowledge and their anxiety response, *B* = 0.016, *SE* = 0.022, *BootCI* (−0.026, 0.059). Notably, its relationship was not statistically significant.

### Moderate Coping Strategies in the Link Between COVID-19 Knowledge and Anxiety

The last objective sought to examine the moderating role of coping strategies in the relationship between COVID-19 knowledge and anxiety.

As presented in Table 5, the results showed that coping strategies generally moderated the relationship between students’ COVID-19 knowledge and their level of anxiety. This moderating role of coping strategies was dynamic and complex depending on the dimensions of the moderator. For example, results on the parallel moderating roles of the coping strategy dimensions revealed that active coping, *B* = −0.056, *SE* = *0.024*, *BootCI* (−0.106, −0.011) and emotional support, *B* = −0.079, *SE* = *0.028*, *BootCI* (−0.134, −0.025) had a significant negative moderating effect on the relationship between students’ COVID-19 knowledge and their level of anxiety. This indicated that high levels of active coping and emotional support coping strategies are required to weaken the positive relationship between COVID-19 knowledge and anxiety of students.

**TABLE 5:** Moderated moderation of coping strategies in the relationship between COVID-19 knowledge and anxiety.

Model		*B*	*SE*	*t*	LLCI	ULCI	*R*^2^ [*F*-value, sig.]	*R*^2^ change [*F*-value, sig.]
1	Constant	0.532	0.260	2.046	0.022	1.042	0.039 [10.303, 0.000]	0.007 [5.775, 0.016]
	Knowledge (KNG)	0.170	0.060	2.810	0.051	0.289		
	Active coping (ACP)	0.375	0.109	3.437	0.161	0.590		
	KNG*ACP	–0.058	0.024	–2.403	–0.106	–0.011		
2	Constant	1.488	0.235	6.320	1.026	1.950	0.013 [3.251, 0.021]	0.001 [0.446, 0.504]
	Knowledge	0.023	0.055	0.412	–0.086	0.131		
	Religious coping (RCP)	–0.065	0.091	–0.710	–0.244	0.115		
	KNG*RCP	0.014	0.021	0.668	–0.027	0.054		
3	Constant	2.138	0.241	8.871	1.665	2.611	0.060 [16.215, 0.000]	0.020 [22.651, 0.000]
	Knowledge (KNG)	–0.179	0.053	–3.356	–0.283	–0.074		
	Behavioral disengagement (BDC)	–0.455	0.125	–3.647	–0.701	–0.210		
	KNG*BDC	0.133	0.028	4.759	0.078	0.187		
4	Constant	0.240	0.288	0.831	–0.326	0.805	0.055 [14.702, 0.000]	0.010 [8.183, 0.004]
	Knowledge (KNG)	0.214	0.065	3.279	0.086	0.342		
	Emotional support (ESS)	0.513	0.126	4.086	0.267	0.760		
	KNG*ESS	–0.079	0.028	–2.861	–0.134	–0.025		
5	Constant	–0.812	0.606	–1.340	–2.002	0.378	0.063 [7.283, 0.000]	0.012 [9.596, 0.002]
	Knowledge (KNG)	0.525	0.147	3.567	0.236	0.814		
	Active coping (ACP)	1.344	0.288	4.674	0.780	1.909		
	KNG*ACP	–0.283	0.066	–4.304	–0.412	–0.154		
	Religious coping (RCP)	0.415	0.266	1.558	–0.108	0.938		
	KNG*RCP	–0.114	0.061	–1.863	–0.234	0.006		
	ACP*RCP	–0.319	0.109	–2.931	–0.533	–0.105		
	KNG*ACP*RCP	0.075	0.024	3.098	0.027	0.122		
6	Constant	–2.676	0.781	–3.426	–4.210	–1.143	0.123 [15.026, 0.000]	0.035 [30.207, 0.000]
	Knowledge (KNG)	0.821	0.183	4.492	0.462	1.180		
	Active coping (ACP)	1.938	0.290	6.678	1.369	2.508		
	KNG*ACP	–0.396	0.066	–6.037	–0.525	–0.267		
	Behavioral disengagement (BDC)	1.783	0.412	4.324	0.973	2.592		
	KNG*BDC	–0.356	0.096	–3.698	–0.545	–0.167		
	ACP*BDC	–0.868	0.149	–5.820	–1.160	–0.575		
	KNG*ACP*BDC	0.186	0.034	5.496	0.120	0.252		
7	Constant	0.624	0.798	0.782	–0.942	2.191	0.060 [6.880, 0.000]	0.000 [0.254, 0.614]
	Knowledge (KNG)	0.123	0.193	0.640	–0.255	0.501		
	Active coping (ACP)	–0.070	0.373	–0.187	–0.801	0.662		
	KNG*ACP	0.022	0.085	0.261	–0.145	0.189		
	Emotional support (ESS)	0.201	0.376	0.536	–0.537	0.939		
	KNG*ESS	–0.023	0.086	–0.265	–0.192	0.147		
	ACP*ESS	0.091	0.152	0.596	–0.208	0.389		
	KNG*ACP*ESS	–0.01	0.034	–0.504	–0.085	0.050		
8	Constant	3.252	0.766	4.248	1.749	4.756	0.068 [7.768, 0.000]	0.002 [1.373, 0.242]
	Knowledge (KNG)	–0.344	0.178	–1.939	–0.693	0.004		
	Religious coping (RCP)	–0.431	0.272	–1.585	–0.965	0.103		
	KNG*RCP	0.067	0.061	1.090	–0.053	0.186		
	Behavioral disengagement (BDC)	–1.089	0.432	–2.519	–1.938	–0.240		
	KNG*BDC	0.235	0.101	2.326	0.037	0.433		
	RCP*BDC	0.234	0.144	1.625	–0.049	0.516		
	KNG*RCP*BDC	–0.038	0.033	–1.172	–0.103	0.026		
9	Constant	–1.049	0.688	–1.525	–2.399	0.301	0.083 [9.671, 0.000]	0.011 [9.072, 0.003]
	Knowledge (KNG)	0.645	0.163	3.958	0.325	0.965		
	Religious coping (RCP)	0.381	0.286	1.331	–0.181	0.942		
	KNG*RCP	–0.138	0.065	–2.107	–0.267	–0.009		
	Emotional support (ESS)	1.459	0.341	4.275	0.789	2.129		
	KNG*ESS	–0.338	0.078	–4.357	–0.491	–0.186		
	RCP*ESS	–0.310	0.128	–2.419	–0.562	–0.058		
	KNG*RCP*ESS	0.086	0.029	3.012	0.030	0.143		
10	Constant	–2.572	0.962	–2.674	–4.460	–0.684	0.130 [16.029, 0.000]	0.023 [19.722, 0.000]
	Knowledge (KNG)	0.878	0.211	4.168	0.464	1.291		
	Behavioral disengagement (BDC)	1.510	0.551	2.739	0.428	2.593		
	KNG*BDC	–0.355	0.121	–2.923	–0.593	–0.116		
	Emotional support (ESS)	2.123	0.382	5.565	1.374	2.872		
	KNG*ESS	–0.464	0.083	–5.593	–0.627	–0.301		
	BDC*ESS	–0.849	0.208	–4.087	–1.257	–0.441		
	KNG*BDC*ESS	0.203	0.046	4.441	0.113	0.293		
11	Constant	1.301	0.213	6.099	0.882	1.720	0.065 [8.681, 0.000]	0.011 [8.931, 0.003]
	Knowledge (KNG)	–0.024	0.031	–0.796	–0.084	0.036		
	Active coping (ACP)	0.127	0.046	2.743	0.036	0.218		
	Religious coping (RCP)	–0.062	0.041	–1.508	–0.142	0.019		
	Behavioral disengagement (BDC)	0.110	0.066	1.682	–0.018	0.239		
	ACP*RCP*BDC	–0.026	0.012	–2.174	–0.049	–0.002		
	KNG*ACP*RCP*BDC	0.006	0.002	2.988	0.002	0.010		
12	Constant	1.171	0.230	5.093	0.720	1.623	0.070 [9.396, 0.000]	0.011 [9.070, 0.003]
	Knowledge (KNG)	–0.045	0.032	–1.422	–0.108	0.017		
	Active coping	0.068	0.048	1.408	–0.027	0.163		
	Behavioral disengagement (BDC)	0.063	0.067	0.950	–0.067	0.194		
	Emotional support (ESS)	0.111	0.049	2.268	0.015	0.207		
	ACP*BDC*ESS	–0.030	0.013	–2.294	–0.056	–0.004		
	KNG*ACP*BDC*ESS	0.007	0.002	3.012	0.003	0.012		
13	Constant	1.668	0.223	7.477	1.230	2.106	0.077 [10.457, 0.000]	0.010 [8.420, 0.004]
	Knowledge (KNG)	–0.016	0.030	–0.527	–0.076	0.044		
	Religious coping (RCP)	–0.125	0.040	–3.097	–0.204	–0.046		
	Behavioral disengagement (BDC)	–0.047	0.065	–0.730	–0.174	0.080		
	Emotional support (ESS)_	0.072	0.049	1.459	–0.025	0.169		
	RCP*BDC*ESS	–0.010	0.011	–0.874	–0.032	0.012		
	KNG* RCP*BDC*ESS	0.006	0.002	2.902	0.002	0.010		
14	Constant	1.287	0.196	6.579	0.903	1.671	0.081 [9.493, 0.000]	0.011 [8.956, 0.003]
	Knowledge (KNG)	–0.017	0.027	–0.632	–0.071	0.036		
	Active coping (ACP)	0.074	0.039	1.890	–0.003	0.151		
	Religious coping (RCP)	–0.095	0.033	–2.912	–0.159	–0.031		
	Behavioral disengagement (BDC)	0.046	0.048	0.961	–0.048	0.140		
	Emotional support (ESS)	0.112	0.041	2.727	0.031	0.192		
	ACP*RCP*BDC*ESS	–0.007	0.003	–2.154	–0.013	–0.001		
	KNG*ACP*RCP*BDC*ESS	0.002	0.001	2.993	0.001	0.003		

Although behavioral disengagement was found to be a significant moderator, *B* = 0.113, *SE* = *0.028*, *BootCI* (0.078, 0.187), it had a positive moderating effect on the relationship existing between the two variables. This suggests that the adoption of the behavioral disengagement coping strategy instead strengthened the positive relationship between COVID-19 knowledge and anxiety. With the exception of three moderators (i.e., Religious coping, KNG*ACP*ESS, and KNG*RCP*BDC) which were non-significant moderators, the rest of the moderators showed a significant positive moderation effect on the relationship existing between COVID-19 related knowledge and anxiety.

## Discussion

This study examined the roles of school climate and coping strategies in the relationship between COVID-19 knowledge and anxiety among Physical Education students from two public universities in Ghana. The findings showed a positive and significant link between COVID-19 knowledge and anxiety, suggesting that a high level of knowledge in COVID-19 is associated with increased levels of anxiety. This finding is contrary to the most common literature-based and logical relationship between COVID-19 related knowledge and anxiety; that persons who are knowledgeable on issues of COVID-19 are expected to demonstrate a low level of anxiety toward issues of COVID-19 (Germani et al., 2020b; Lin et al., 2020; Li and Lyu, 2021; Šrol et al., 2021). This counterintuitive finding appeared surprising since adequate knowledge in COVID-19 rather led to heightened levels of anxiety and that students reported being more anxious as they got to know more about COVID-19. The COVID-19 pandemic brought about unparalleled fear, apprehension and nervousness such that getting more knowledge on the seriousness of the virus elicited anxiety-related experiences (Gong et al., 2020). Additionally, the Centers for Disease Control and Prevention [CDC] (2020) reported that regardless of people’s level of awareness, the COVID-19 pandemic has caused fear and anxiety among people since its outbreak.

Due to the unfolding events after the virus outbreak, students may subsequently feel vulnerable or susceptible within the school and classroom environments. For Physical Education students, their practical sessions usually expose them to intermittent contact or interaction with other colleagues. This instructional set, to some extent, may create a perceived unsafe learning climate on the possibility of contracting the virus for more knowledgeable students. Comparatively, those who were less knowledgeable on the perceived susceptibility and severity of the virus were less anxious. Hence, once students are knowledgeable about COVID-19, they become aware of the consequences of contracting the virus. The awareness of the virulence and case fatalities may generate feelings of anxiety among students. Everyone become suspicious of others, and this scenario might lead to fear, and subsequently increased levels of anxiety and other psychological tensions (Dong and Bouey, 2020; Shigemura et al., 2020; Wolf et al., 2020). Alternatively, the positive relationship between COVID-19 knowledge and anxiety can be heightened when this knowledge is exaggerated. In the observations of Rajkumar (2020), COVID-19 may trigger anxiety, particularly when the information is either exaggerated or inaccurate. Other studies have obtained findings that reflect this claim (see Centers for Disease Control and Prevention [CDC], 2020; Du et al., 2020; Fardin, 2020; Lee and You, 2020; Li et al., 2020c; Sorokowski et al., 2020; Wang et al., 2020; Zhai and Du, 2020). It is important to mention that knowledge is necessary for COVID-19. Scholars like Krawczyk et al. (2013) and Yıldırım et al. (2020) found that having adequate knowledge during an epidemic or disease-related literacy of people in any population plays a central role in the adoption of preventive behaviors. Whereas knowledge is very crucial in helping people protect themselves, there is the need for practitioners to understand the context, procedure, and platforms with which education or information about COVID-19 should be provided to persons.

This finding, however, contradicts a previous study which found that knowledge on COVID-19 was not associated with anxiety (Lin et al., 2020). The variations noted in the current study and that of Lin et al. (2020) could be attributed to the use of the general population as against university students. The variability in the general population may have confounded the findings of this study. The practical implication of this finding is that further increasing COVID-19 awareness or campaign programs could raise peoples’ intentions to adopt preventive behaviors against diseases as a result of their heightened anxiety levels.

The findings of the study also showed that school climate moderates the link between COVID-19 knowledge and anxiety. Among the students that reported a positive school climate, the effect of knowledge on anxiety was negative, however, this effect was positive for students that reported a positive school climate. The results generally imply that with the same level of COVID-19 knowledge advancement, students that reported positive school climate experienced decreased anxiety, whereas those with the negative school climate experienced an increased level of anxiety. It can therefore be said that the conduciveness of conditions in schools during this COVID-19 era, to a large extent, has a great impact on the management of COVID-19 related anxieties (Germani et al., 2020b; Li and Lyu, 2021; Šrol et al., 2021). Thus, a positive school climate buffers the positive effect of knowledge on anxiety (Lin et al., 2020). The implication is that the relationship between COVID-19 knowledge and anxiety is contingent on school climate. Therefore, the provision of a protective school environment and ensuring the safety of the school environment are necessary steps to reduce COVID-19 related anxieties. When the school environment is safe, even when students have advanced knowledge on COVID-19, they are more likely to feel some sense of inner security and this would lead to low anxiety. This finding emphasizes the need for the provision of a safe and supportive school environment which should largely be provided by educators, parents and the school institution itself (UNESCO, 2020; Laar et al., 2021).

It was further identified that three of the four dimensions of coping, namely active, emotional support, and behavior disengagement coping were significant moderators of the relationship between COVID-19 knowledge and anxiety. While active and emotional support decreased the effect of COVID-19 knowledge on anxiety, behavior disengagement rather increased the effect. The nature of the moderations is such that, with a high level of COVID-19 knowledge, the high adoption of active and emotional coping strategies reduced the level of anxiety among students. This finding has been confirmed by previous research (Iddi et al., 2021; Lin et al., 2020) by indicating that when students highly adopt active coping mechanisms such as taking direct actions to get around problems, making conscious efforts in doing something, and approaching issues step-by-step, among others, would act as buffers in reducing the anxiety related to COVID-19. Similarly, when students employ emotional support coping strategies such as discussing feelings with others, getting sympathy and emotional support from significant others (e.g., teachers, parents or family relations), they are more likely to deal with their fears and nervousness. These approaches may lead to a reduction in their level of anxiety, as supported by recent studies (Jungmann and Witthöft, 2020; Labrague, 2021). However, on the part of behavior disengagement coping, its introduction in the relationship rather increased anxiety as expected. Hence, disengagement coping is dysfunctional and counterproductive because it involves quitting, giving up, and not putting in much effort in an attempt to solve a problem.

Religious coping did not significantly moderate the relationship between COVID-19 knowledge and anxiety. This implies that putting one’s trust in God and seeking help from an object of worship did not necessarily matter as far as the relationship between COVID-19 knowledge and anxiety is concerned. This finding could be as a result of the religious bodies’ active engagement in the propagation of COVID-19 related advocacies such as wearing of nose masks, washing of hands at regular intervals, practicing social distancing, using sanitizers, among others within the Ghanaian context. Generally, these practices appear to be more of active coping than religious coping. Another possible reason for this finding could be due to the sample drawn for this study. The study used Physical Education students who as of the time of data collection had not contracted COVID-19 (not clinically diagnosed), possibly with the belief that taking measures to prevent the contraction of the disease is key.

Interestingly, when religious coping was paired with other coping strategies such as active coping, emotional support, and behavior disengagement in six different instances, the moderation was significant for five out of the six. This identification suggests that religious coping on its own does not alter the relationship between COVID-19 knowledge and anxiety unless it is paired with other coping mechanisms. When paired with other coping strategies, they jointly have a positive impact on anxiety. Thus, they increase levels of anxiety, with an increasing level of knowledge. Even though active coping solely had a negative impact on anxiety, when paired with other forms of coping strategies, it had a positive impact on anxiety, suggesting that the ideal negative effect of active coping was subdued by other coping strategies. This finding suggests that to reduce the anxiety levels during COVID-19 among students, active coping should be predominantly employed. Summarily, the current study adds to the existing literature in that school climate and coping strategies were identified as confounding variables that could affect the link between COVID-19 knowledge and anxiety. This information provides more clarity on the findings compared to previous studies, especially those that only examined the uni-directional relationship.

### Limitations and Future Directions

Despite the strengths of using a relatively large sample size and generating interesting findings from quantitative analysis of empirically verifiable data, this study is not devoid of some limitations. The most prominent limitation is that the data were collected via a single or snapshot assessment as opposed to momentary assessment through alternative methods of data collection like the experience sampling method (ESM) or the ecological momentary assessment (ECA) to generate an idiographic profile that mirrors the dynamic nature of COVID-19. Future studies could adopt longitudinal designs where study participants may be required to record their reported anxiety experiences and associated coping options in a diary process format. The cross-sectional nature of the study makes it impossible to draw causal inferences among identified patterns and other social desirability concerns such as selection biases. The study did not have participants who have either been infected with the virus at the time of data collection or had recovered from COVID-19. Therefore, the findings of this research may not apply to such populations. The findings of this study can be applied to other program areas, such as Music or Theatre Arts, and Vocational Technical Education, which has practical lessons as part of the program. This notwithstanding, generalizing the findings from the study to other populations should therefore be done with caution. Future studies should also cover the general student population in higher education using similar variables. Through detailed exploration, some variables such as gender, COVID-19 test status (i.e., positive or negative), source of information on COVID-19, among others, can help explain the relationship between COVID-19 knowledge and anxiety among students.

## Conclusion

This research showed a counterintuitive relationship between students’ COVID-19 knowledge and the associated anxiety response. This highlights the notion that educating persons about COVID-19 related matters does not alone help with reducing anxiety response, especially for students. Present findings provide evidence that further increasing COVID-19 awareness or campaign programs could raise peoples’ intentions to adopt preventive behaviors, should not be the primary motive for educators, higher educational institutions, administrators, parents, media, and governments. Instead, a positive school climate (i.e., a protective school environment, free from potential threats with lower chances of getting infected with COVID-19) should be created by stakeholders. Students should be given the necessary interventions or platforms to learn and demonstrate appropriate coping strategies. Educational institutions should create a conducive environment safe for teaching and learning, especially for practical oriented programs like PE during the ongoing pandemic. Particularly, seminars and orientation programs should be developed by school counselors, school healthcare workers, and institution administrators to help students adopt functional active coping strategies, including emotional support.

## Data Availability Statement

The raw data supporting the conclusions of this article will be made available by the authors, without undue reservation.

## Ethics Statement

The studies involving human participants were reviewed and approved by the Institutional Review Board of the University of Cape Coast, Ghana, with a reference number: UCCIRB/EXT/2020/25. The participants provided their written informed consent to participate in this study.

## Author Contributions

JH conceived the idea. FQ performed the analysis. FQ, JH, FA, JF, MS-S, FS, and TS prepared the initial draft of the manuscript. All authors thoroughly revised and approved the final version of the manuscript.

## Conflict of Interest

The authors declare that the research was conducted in the absence of any commercial or financial relationships that could be construed as a potential conflict of interest.

## Publisher’s Note

All claims expressed in this article are solely those of the authors and do not necessarily represent those of their affiliated organizations, or those of the publisher, the editors and the reviewers. Any product that may be evaluated in this article, or claim that may be made by its manufacturer, is not guaranteed or endorsed by the publisher.
