# Supplementary material for: Relationship Between COVID-19 Related Knowledge and Anxiety Among University Students: Exploring the Moderating Roles of School Climate and Coping Strategies
Source: Front Psychol. 2022 Mar 30;13:820288. doi: 10.3389/fpsyg.2022.820288 (PMC9007403; doi:10.3389/fpsyg.2022.820288)
Supplement: Supplementary file 1 [file Data_Sheet_1.pdf]

PSYCHOSOCIAL AND SCHOOL CLIMATE COVID-19 SURVEY QUESTIONNAIRE

Dear respondent

We are a group of researchers, conducting a study on the psychosocial and work climate responses or experiences of PE students toward the coronavirus pandemic. The rationale of this study is to investigate personal responses or experiences in the teaching of PE and Sport amidst the COVID-19 pandemic. You are assured of anonymity and confidentiality as your participation in this study would pose no likely threat to you, now and in the future. Please answer each question as accurately as possible by ticking or indicating your response in the spaces provided. There are no right or wrong answers.

For any information about this research, contact the following; Dr. John Elvis Hagan Jr.: elvis.hagan@ucc.edu.gh and Mr. Francis Sambah: francis.sambah@stu.ucc.edu.gh.

Please do you consent to participate in this study?

Yes O

No O

1. What is your age?
   a. 20-24 O
   b. 25-29 O
   c. 30-34 O
   d. 35-39 O
   e. 40 and above O
2. What is your sex?
   a. Male O
   b. Female O
3. What is your religion?
   a. Christian O
   b. Muslim O
   c. Traditionalist O
   d. Others O

School Climate

This section elicits responses on PE students' school climate relative to COVID-19. Your sincere and genuine responses would be required. Kindly tick or indicate your responses at the required box or spaces provided.

4. Do you feel confident participating in practical lessons during the COVID-19 outbreak?

   O Yes

   O No
5. Are the necessary protective equipment available for practical lessons amidst the outbreak of COVID-19?

   O Yes

   O No
6. Are you comfortable participating in teaching and learning activities amidst this COVID-19 outbreak?

   O Yes

   O No
7. Have you been educated on how to protect yourself during practical lessons in COVID-19 times?

   O Yes

   O No
8. Is the practical lesson environment safe enough to prevent infections and transmission of COVID-19?

   O Yes

   O No

Anxiety Scale

Below is a list of common symptoms of anxiety. Please carefully read each item in the list and indicate how much you have been bothered by that symptom during the past month in relation to COVID-19, including today, by ticking the number in the corresponding space in the column next to each symptom.

0 = Not at All

1 = Somewhat

2 = Moderately

3 = Very much so

9. I feel relaxed	0	1	2	3
10. I am very concerned	0	1	2	3
11. I feel unsteady	0	1	2	3
12. I feel nervous	0	1	2	3

13. I have self-doubts 0 1 2 3

14. I fear the worst happening 0 1 2 3

PE Students' Knowledge of COVID-19

This section elicits responses on knowledge about SARS-CoV-2, the virus responsible for COVID-19 pandemic. Your sincere and genuine responses would be required. Kindly tick or indicate your responses at the required box or space provided.

15. Treatment of coronaviruses focuses on managing symptoms as the virus runs its course?

    O True

    O False

16. Do health measures such as early case detection and isolation, contact tracing and isolation, and social distancing help reduce the spread of COVID-19?

    O Yes

    O No

17. The emergence of asymptomatic carriers is the major challenge to the control of COVID-19 pandemic?

    O Yes

    O No

18. Finding infectious patients and cutting off transmission routes are important methods to the control of COVID-19?

    O Yes

    O No

19. At present, is herd immunity possible for recovered COVID patients against reinfection?

    O Yes

    O No

20. What are the main ways in which people are currently getting infected with the new coronavirus?

    Please select one response option only.

    O Eating or touching bats

    O Fecal contaminants in drinking water

    O Unhygienic preparation of food

    O Sexual intercourse or sharing of needles for drug use

    O Droplets of saliva that land in the mouths or noses of people who are nearby

O Eating undercooked meat products

Coping Strategies toward COVID-19

This section focuses on how people respond to difficult or stressful events in their lives. Different events bring out somewhat different responses, but think about what you usually do when you are under stress induced by the COVID-19 pandemic. There are no "right" or "wrong" answers.

Instructions: Indicate your experience with stressful events related to COVID-19 pandemic by ticking the number that correspond to your response using the response set below.

1 = **Not**
2 = **Somewhat or moderately so**
3 = **much**
4 = **Very much**

Active coping

21. I concentrate my efforts on doing something about it.	1	2	3	4
22. I take additional action to try to get rid of the problem.	1	2	3	4
23. I take direct action to get around the problem.	1	2	3	4
24. I do what has to be done, one step at a time.	1	2	3	4

Religious coping:

25. I put my trust in God/object of worship	1	2	3	4
26. I seek the help of my object of worship	1	2	3	4
27. I try to find comfort in my religion.	1	2	3	4
28. I pray more than usual.	1	2	3	4

Behavioral disengagement:

38. I admit to myself that I can't deal with it, and quit trying	1	2	3	4
39. I just give up trying to reach my goal.	1	2	3	4
40. I give up the attempt to get what I want	1	2	3	4
41. I reduce the amount of effort I put into solving the problem	1	2	3	4

Use of emotional support:

42. I discuss my feelings with someone	1	2	3	4
43. I try to get emotional support from friends or relatives.	1	2	3	4
44. I get sympathy and understanding from someone.	1	2	3	4
45. I learn to live with it.	1	2	3	4

Thank you
